# Wastewater-based epidemiological study on helminth egg detection in untreated sewage sludge from Brazilian regions with unequal income

**DOI:** 10.1186/s40249-025-01314-8

**Published:** 2025-06-11

**Authors:** Andrey Duarte Boava, Alberto Jorge da Rocha Silva, Paula Beatriz Santiago, Bruno Dias Batista, Daniela Cunha Coelho, Ivana Mirtes Feu Silva, Patrícia Barbosa Machado, Patrícia Souza Sobrinho, Carla Nunes de Araújo, Izabela Marques Dourado Bastos

**Affiliations:** 1 Pathogen-Host Interface Laboratory, Department of Cell Biology, University of Brasília, Brasília, Brazil; 2 Coordination of Biological and Limnological Analyses, Environmental Sanitation Company of the Federal District, Brasília, Brazil

**Keywords:** Helminthiases, Wastewater, Environmental surveillance, One Health, Public health, Brazil

## Abstract

**Background:**

Helminthiases are neglected diseases that affect billions of people worldwide, particularly those with inadequate sanitation, poor hygiene practices, and limited access to clean water. Due to frequent underreporting, wastewater-based epidemiology has emerged as a valuable tool for monitoring parasitic infections at population-level. This study aimed to detect and quantify helminth eggs in untreated sewage sludge from eight wastewater treatment plants located in different Brazilian socioeconomic regions.

**Methods:**

The study was conducted from June 2021 to December 2023 in Goiás and Federal District, the Brazilian federative unit with the highest income inequality. Samples were collected bimonthly (*n* = 121). Helminth eggs were recovered using centrifugation and flotation with a ZnSO_4_ solution (d = 1.30 g/ml). After 21–28 days of incubation in sulfuric acid, viable eggs were identified and counted using a Sedgewick-Rafter Chamber under an optical microscope. Statistical analyses included One-way analysis of variance (ANOVA) followed by Tukey’s multiple comparisons test to evaluate differences in helminth egg counts between low-, medium- and high-income regions.

**Results:**

Twelve helminth genera were identified, revealing significant differences in prevalence and diversity across socioeconomic strata. Cestode eggs, particularly *Hymenolepis* spp. (44.28%), were the most prevalent overall. Trematode eggs were less frequent but exhibited greater taxonomic diversity. Sludge from low-income areas had the highest egg concentration [16.61 ± 3.02 eggs per gram of dry mass ( eggs/g DM)], nearly five times greater than in high-income areas such as Brasília Norte (3.56 ± 0.55 eggs/g DM; *P* = 8.8 × 10⁻⁹). *Ascaris* spp. (19.27%) and *Trichuris* spp. (7.90%) predominated in low-income areas. Medium-income regions showed intermediate values, with notable regional variation.

**Conclusions:**

Our results demonstrate that helminth egg diversity and concentration in sewage sludge are closely related to the socioeconomic characteristics of the served population. These findings may inform prevention and control strategies in vulnerable areas and support the development of public health and sanitation policies that address social and environmental inequalities in Brazil’s Central-Western region.

**Graphical abstract:**

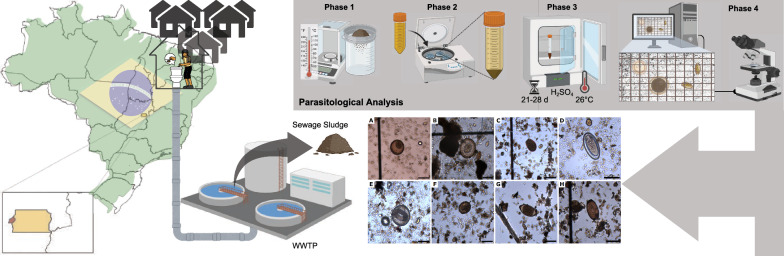

## Background

Helminths are parasitic worms that can be transmitted to humans through multiple routes. Soil-transmitted helminths (STH; e.g., *Ascaris lumbricoides*, *Trichuris trichuris*, and hookworms) are acquired mainly by fecal-oral route, by the ingestion of eggs present in contaminated soil, water, or food. Another form of transmission is the active skin penetration of the infective stage larvae when people walk barefoot, particularly for hookworms and *Strongyloides stercolaris*. Other helminth species, such as *Schistosoma* spp., *Hymenolepis* spp., and *Diphyllobothrium* spp., can be transmitted through arthropod vectors, which act as intermediate hosts in the parasite’s life cycle [1].

According to the World Health Organization [2], STH infections affect more than 1.5 billion people worldwide, particularly in underdeveloped and developing countries, highlighting the socio-economic factors involved [3]. They are primarily associated with inadequate sanitation, insufficient access to treated water, and close contact with domestic animals [4]. Brazil has one of the highest prevalences of STH infections in the Americas, affecting over 10.9 million people across all regions of the country, especially in at-risk populations, such as indigenous and school-aged children [5–7]. Moreover, STHs are commonly neglected as a cause of death in Brazil, primarily due to the non-mandatory notification of cases. The most common species responsible for STHs include *A. lumbricoides*, whipworm (*T. trichiura*), *S. stercoralis*, and hookworms (*Ancylostoma duodenale* and *Necator americanus*) [4]. These species are usually grouped due to their similar diagnostic procedures and response to the same medications.

Initially asymptomatic or presenting mild and nonspecific symptoms, helminth infections can progress to moderate to high-intensity cases, causing gastrointestinal symptoms and nutritional deficiencies [8]. Among them, ascariasis is the most prevalent globally, affecting between 0.7 and 1.2 billion people [9, 10]. One likely factor associated with the prevalence of this infection is the remarkable resistance of *Ascaris* spp. eggs to commonly adopted sewage treatments. These eggs have a thick, multilayered shell that provides protection against extreme temperatures, desiccation, and chemical disinfectants commonly used in wastewater treatment, allowing them to persist in the environment for long periods and facilitating transmission [11, 12]. In Brazil, ascariasis, trichuriasis, and hookworm infections remain widespread and frequently occur in coinfections [13].

Wastewater treatment plants (WWTPs) generate tons of solid waste each year, including sewage sludge, a byproduct of the wastewater treatment process. Sewage sludge monitoring is a practice used to identify and quantify helminth eggs, assess the parasite burden, and estimate the prevalence of parasitic infections in specific populations. During the SARS-CoV-2 pandemic, wastewater-based epidemiology (WBE) has gained prominence as a method to evaluate viral levels in sewage systems across Brazil [14–16] and several other countries, including the United Kingdom [17], United States [18] and France [19]. Despite its recent visibility, this technique has been employed for over a decade for various purposes, such as monitoring the consumption of legal and illegal drugs [20, 21], and pathogen levels during other pandemics [22]. In Brazil, the Federal District stands out as the most unequal Federated Unit [23], with both the largest slum and the wealthiest neighborhood in the country coexisting within its territory [23, 24]. This study aimed to perform a parasitological analysis of sewage sludge from WWTPs in the Federal District and neighboring Goiás state to identify and quantify helminth eggs across regions with different socioeconomic profiles. Although previous Brazilian studies using WBE have detected helminth in sewage, few have explored regional variations or their association with social inequality [25, 26]. This study aims to bridge this gap and provide data to inform public health and sanitation strategies.

## Methods

### Study area and sewage sludge sampling

The Federal District, located in the Center-West Region of Brazil, has an estimated population of 2,817,381 people and is divided into 35 administrative regions [24]. It is the smallest Brazilian federative unit, covering approximately 0.6% (5,761 km^2^) of the national territory [24]. The Environmental Sanitation Company of the Federal District (CAESB) is responsible for sanitation services in all the administrative regions and the city of Águas Lindas de Goiás(AGL). There are 17 WWTPs in operation, eight of which periodically monitor untreated sewage sludge [27]. Seven are situated in different administrative regions within the Federal District, and one is in AGL. The detailed specifications for each WWTP, including the average flow rate, treatment methods employed, and population served by each facility, are provided in Table 1.

**Table 1:** Operational characteristics of wastewater treatment plants (WWTPs), population equivalents, and socioeconomic profiles of served regions in the Federal District and Goiás neighboring state (2023)

WWTP	Average flow rate (L/s)	Biological treatment	Population (in thousands)	Average monthly income (MW)
Águas Lindas de Goiás (AGL)	99.1	UASB reactor + CMAL + Aerated facultative lagoon	103.4	0.7
Brasília Norte (BSN)	600.0	Activated sludge (modified Bardenpho process)	313.0	4.1
Brasília Sul (BSS)	1,232.1	Activated sludge (modified Bardenpho process)	528.3	5.0
Gama (GAM)	185.6	UASB reactor + activated sludge	90.0	1.6
Melchior (MLC)	1,288.7	UASB reactor + aerobic reactor (UNITANK®)	711.4	2.4
Recanto das Emas (RCE)	155.6	UASB reactor + CMAL + Facultative Lagoon	118.5	1.2
Riacho Fundo (RF1)	80.9	Activated sludge (batch operation)	52.4	1.9
Sobradinho (SB1)	115.9	Activated sludge	78.4	1.5

UASB: Upflow Anaerobic Sludge Blanket; CMAL: Complete-Mix Aerated Lagoon. MW: minimum wage. The data are from CAESB [27] and Codeplan (GDF) [28]. The population was estimated by CAESB based on the organic load, utilizing the 2023 average influent Biochemical Oxygen Demand (BOD) and the standard value of 0.054 kg BOD/person/day [29]. The average monthly income was derived from the mean of all administrative regions served by each WWTP, considering the ratio between the average household per capita incomes reported by Codeplan in 2021 and the prevailing minimum wage for that year (BRL1100.00)

Based on the analyses provided by the Institute of Research and Statistics of the Federal District (IPEA) [28], the WWTPs can be classified into three categories according to the average income of the areas they serve: (I) low-income, which includes AGL; (II) medium-income, comprising Gama (GAM), Recanto das Emas (RCE), Melchior (MLC), Riacho Fundo (RF1), and Sobradinho (SB1); and (III) high-income, which includes Brasília Norte (BSN) and Brasília Sul (BSS).

The sewage sludge samples were analyzed bimonthly between June 2021 and December 2023 (*n* = 121), except when the WWTPs were not operational. Samples were collected following the methodology described in the ABNT/NBR 10007 standard [30]. The solid samples were placed in 710 ml sterilized sample collection bags Whirl–Pak® (Nasco, Fort Atkinson, WI, USA), correctly identified, and filled to at least half of their capacity. All samples were collected before thermal treatment for pathogen removal, and transportation from WWTPs to the laboratory was conducted using appropriately refrigerated thermal polystyrene boxes.

### Sample preparation

**Fig. 1:**
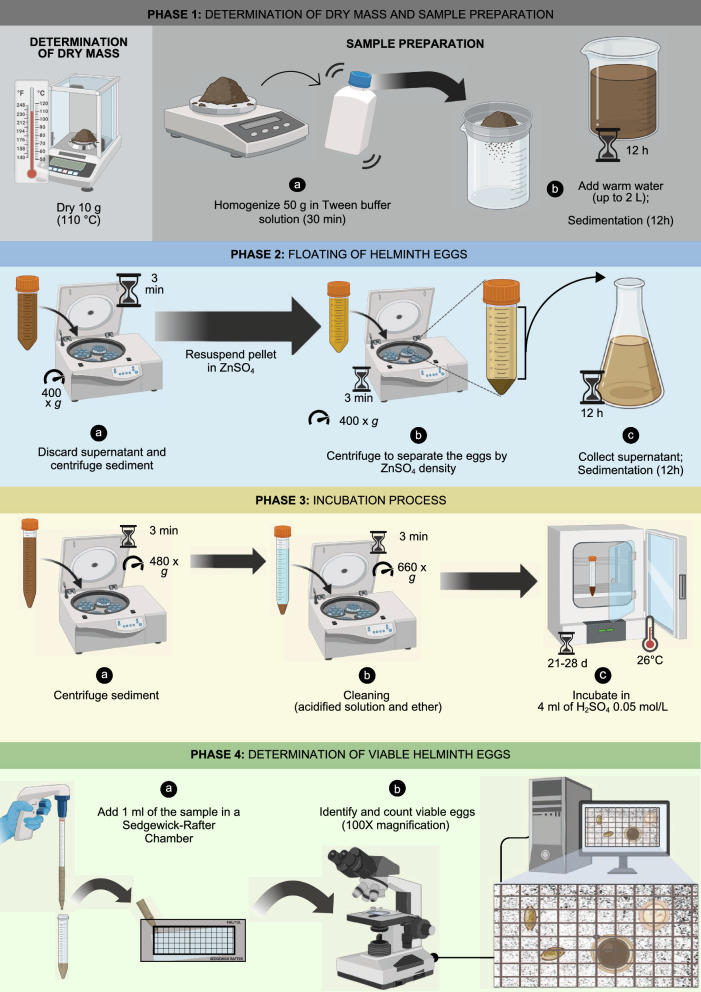
Schematic illustration of the methodology proposed by Yanko [31] and modified by Thomaz-Soccol et al. [32]. Sedimentation, centrifugation, and flotation (phases 1, 2, and 3) of helminth eggs in zinc sulfate solution (d = 1.30 g/ml) are illustrated. The eggs were identified and counted by microscopic analysis using a Sedgewick-Rafter chamber (phase 4). Figure created with BioRender.com

The procedure followed the method proposed by Yanko [31] with modifications from Thomaz-Soccol et al. [32], as outlined in Fig. 1. Composite samples were dried to determine dry mass, then homogenized in a 450 ml buffer solution [Tween 80 0.1% (v/v); KH_2_PO_4_ 0.25 mol/L pH 7.2; MgCl_2_ 0.4 mol/L] and left to settle. After a 12-h sedimentation, the pellets were transferred to 50 ml tubes and centrifuged at 400 × *g* for 3 min. Supernatants were discarded, and the pellets were resuspended in 50 ml of ZnSO_4_ solution (d = 1.30 g/ml) and centrifuged at 400 g for 3 min. After further washing and sedimentation, samples were centrifuged at 480 × *g* and finally transferred to 15 ml tubes. Pellets were then treated with an acidified alcoholic solution [CH_2_CH_3_OH 35% (v/v); H_2_SO_4_ 0.05 mol/L] and 3 ml of diethyl ether, vortexed, and centrifuged at 660 × *g* for 3 min. This was repeated until supernatants were clear. Final pellets were resuspended in 4 ml of 0.05 mol/L H_2_SO_4_ solution and incubated at 26 ± 1 ºC for 21–28 days.

### Determination of viable helminth eggs

After incubation, the samples were homogenized, and 1 ml was added to the Sedgewick-Rafter Chamber. The identification and counting of viable helminth eggs were performed under an optical microscope (Axio Scope.A1, ZEISS, Oberkochen, Germany) at 100 × magnification. Images were recorded by a digital camera attached to the equipment (Axiocam 506 Color, ZEISS, Oberkochen, Germany) and its corresponding software (ZEN 3.1 Blue Edition, ZEISS, Oberkochen, Germany).

The results of the parasitological analysis, expressed as the number of viable eggs per gram of dry mass (eggs/g DM), were calculated according to Eq. 1:

1 $${\text{N}}_{{{\text{eggs}}}} /{\text{g DM }} = \frac{{{\text{Ne }} \times {\text{ Vc }} \times {\text{ Vf}}}}{{{\text{Ap }} \times \%{\text{ DM}}}}$$

Where: N_eggs_/g DM = number of viable helminth eggs per gram of dry mass; Ne = number of viable helminth eggs counted across the sample; Vc = capacity of Sedgewick-Rafter Chamber (1 ml); Vf = final volume of the incubated sample (4 ml); Ap = sample mass processed in the analysis (50 g); and % DM = percentage of the total dry mass, calculated from the ratio between the final dry mass indicated by the equipment and the sample mass weighed before the process (~ 10 g). The density of the water and H_2_SO_4_ solution was 1 g/ml for calculation purposes.

### Statistical analyses

All analyses were performed using GraphPad Prism^®^ software version 8.4.0 (GraphPad Software Inc.; San Diego, CA, USA) and *P* values < 0.05 were considered statistically significant (95.0% confidence level). Data are presented as the mean ± standard error of the mean (SEM). One-way analysis of variance (ANOVA) was used to compare the number of viable helminth eggs per gram of dry mass (eggs/g DM) across different WWTPs serving low-, medium-, and high-income regions, followed by Tukey’s multiple comparisons test to identify statistically significant differences between them.

## Results

### Parasitological profile

**Fig. 2:**
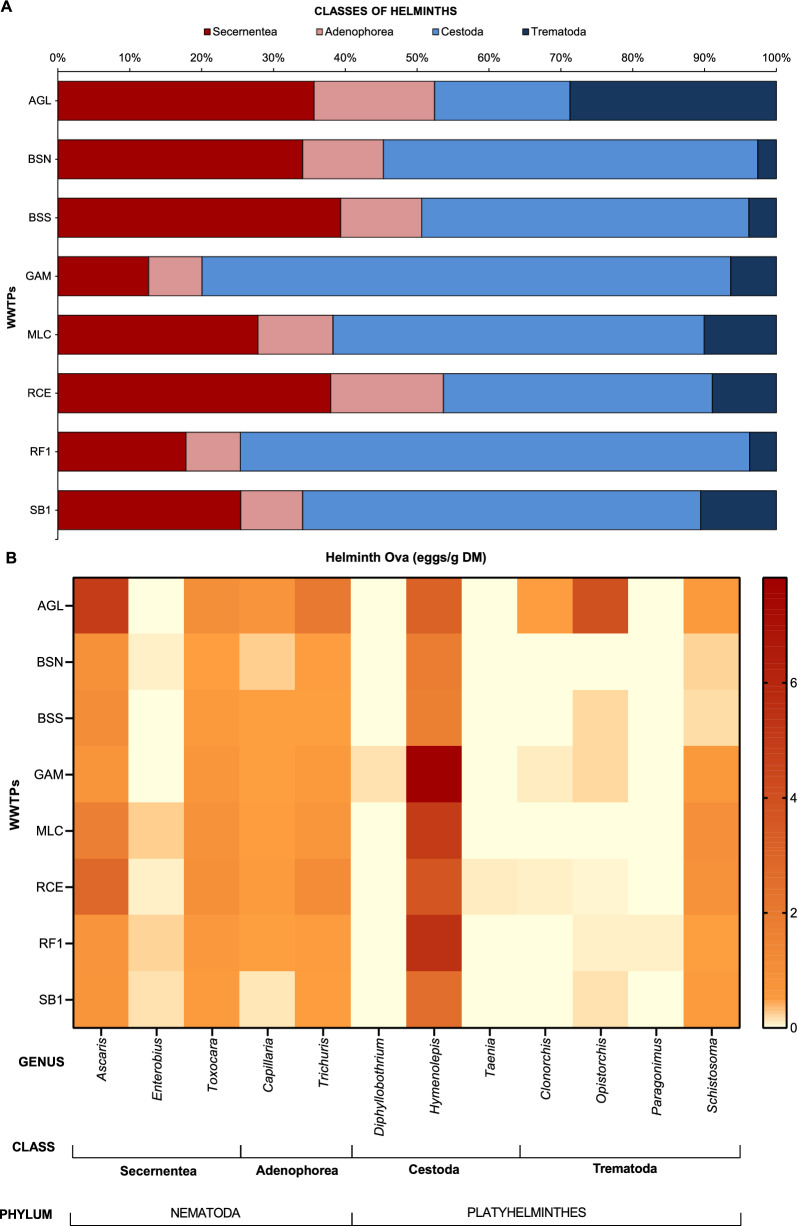
Parasitological profile of helminth egg classes found in the sewage sludge of each analyzed wastewater treatment plant (WWTP) (**A**) and a heatmap (**B**) showing the average number of helminth ova detected in each WWTP (2021–2023) by genus

After microscopic analysis, helminth eggs from the phyla Platyhelminthes and Nematoda were identified. Cestodes (phylum Platyhelminthes) were the most abundant group in the samples analyzed, except for AGL and RCE WWTPs (Fig. 2A), that serve primarily low- and medium-income populations, where Secernentea (phylum Nematoda) eggs were predominant [5.92 eggs/g DM (35.65%), and 3.83 eggs/g DM (37.99%), respectively]. In other WWTPs, the predominance of cestode eggs is primarily attributed to the high presence of eggs from the genus *Hymenolepis* (44.28%; 31.35 eggs/g DM), distinguishing their parasitological profile from those mentioned above. AGL, which serves low-income regions, exhibited a different parasitological profile compared to the WWTPs in the Federal District (medium- and high-income areas, which showed a very similar profile), mainly due to the higher presence of *Ascaris* sp. (4.91 eggs/g DM), *Trichuris* sp. (2.04 eggs/g DM) and *Opisthorchis* sp. (3.95 eggs/g DM) eggs (Fig. 2B). Notably, the most prevalent nematode species in this WWTP are highly resilient to environmental factors and are commonly associated with poor sanitation practices and inadequate waste management.

Trematode eggs were the least abundant in the analyzed samples (9.35%; 1.04 eggs/g DM). Despite their low occurrence, the class presented the most significant number of identified genera, including *Schistosoma* sp., *Clonorchis* sp., *Opistorchis* sp., and *Paragonimus* sp. eggs. The Adenophorea nematoda class also demonstrated a low prevalence in all the analyzed WWTPs (11.13%; 1.00 egg/g DM), with two genera identified: *Trichuris* and *Capillaria*. Overall, there was an almost equal incidence of nematodes (50.51%; 4.42 eggs/g DM) and platyhelminths (49.49%; 4.11 eggs/g DM) in AGL, BSN, BSS, and RCE, with a predominance of platyhelminth eggs in GAM (79.93%; 8.57 eggs/g DM), MLC (61.70%; 5.95 eggs/g DM), RF1 (74.61%; 5.81 eggs/g DM), and SB1 (65.93%; 3.04 eggs/g DM).

**Fig. 3:**
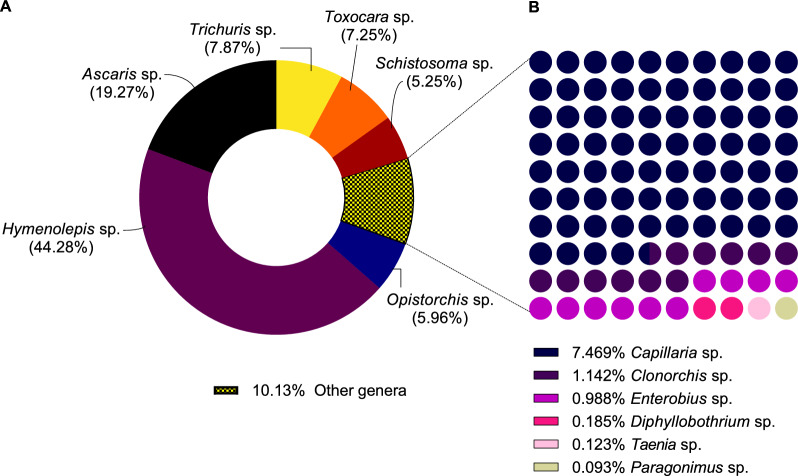
The taxonomy identification of helminth eggs found in the studied regions indicates the most prevalent (**A**) and least frequent (**B**) helminth eggs

**Fig. 4:**
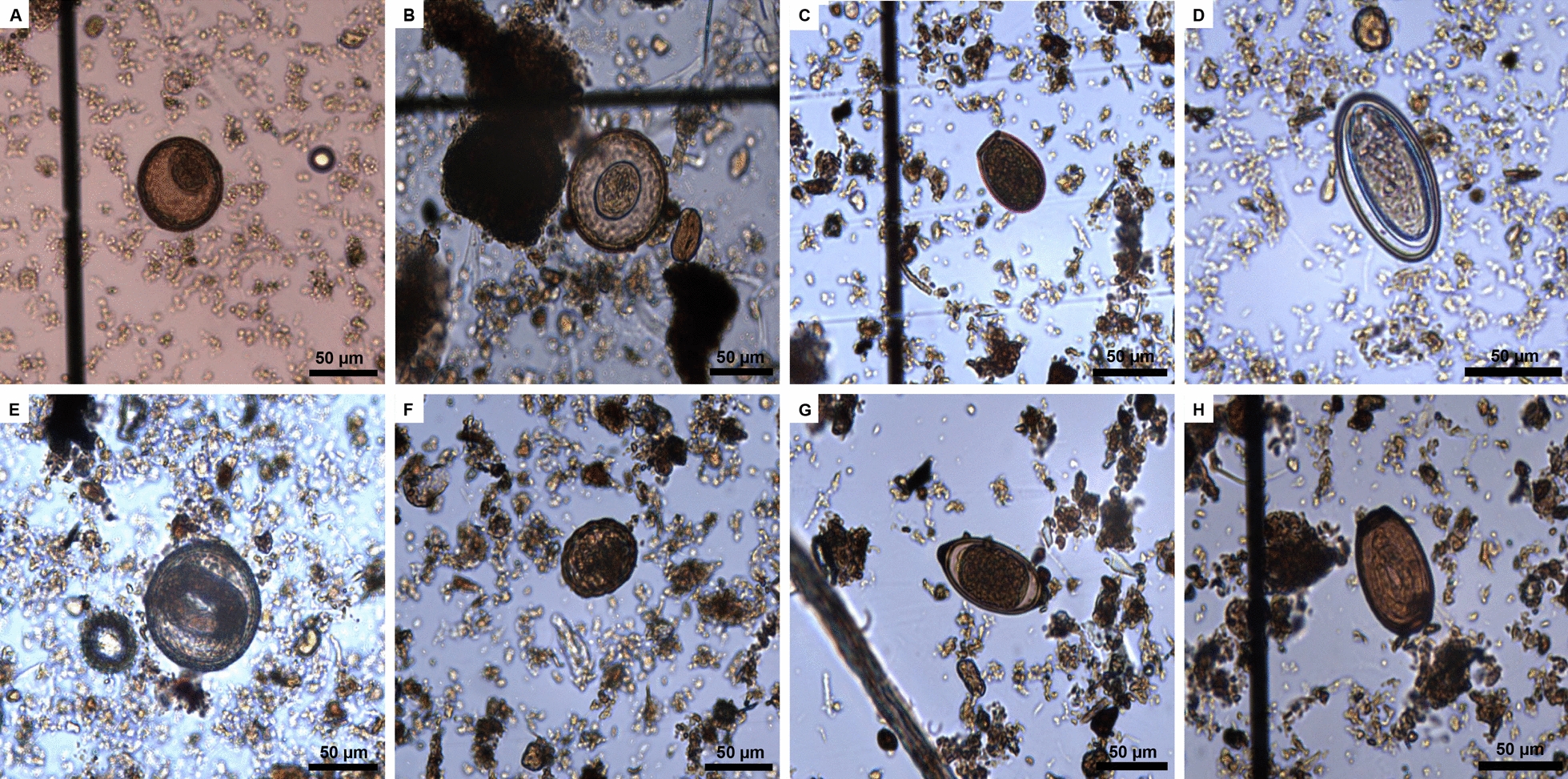
Light microscope photographs of helminth eggs identified in sewage sludge samples: (**A**) *Hymenolepis diminuta*; (**B**) *H. nana*; (**C**) *Opistorchis* sp.; (**D**) *Enterobius vermicularis*; (**E**) Ascaris* lumbricoides*; (**F**) *Toxocara* sp. (larvated); (**G**) *Trichuris trichiura*; (**H**) *Capillaria* sp. (larvated). Scale bars: 50 µm

Eggs from twelve different genera of helminth were identified in the eight WWTPs (Fig. 3). The cestode *Hymenolepis* was the most prevalent in the samples, constituting nearly half (44.28%) of all helminth eggs observed (Fig. 3A). Helminth ova from the two main species of the genus, *H. nana* and *H. diminuta*, were identified (Fig. 4A, B). Viable eggs of *Ascaris* sp. also constituted a significant portion (19.27%) of the identified eggs, standing out as the most abundant nematode (Fig. 3A). Therefore, the analysis of sewage sludge suggested a prevalence of hymenolepiasis and ascariasis within the population served by the analyzed WWTPs. Eggs from specific flatworm groups, including *Taenia* sp., *Diphyllobothrium* sp., and *Paragonimus* sp., were less frequent, suggesting a lower prevalence in the Federal District population (Fig. 3B).

### Helminth load

**Fig. 5:**
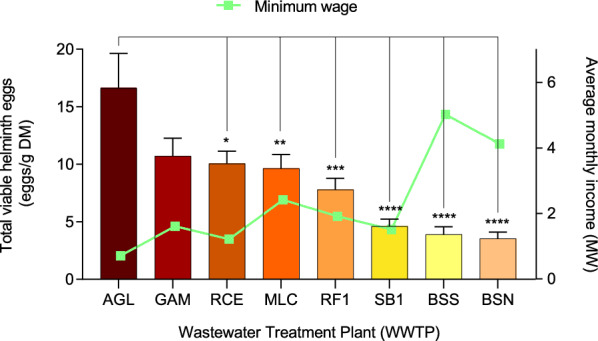
Relationship between average monthly income and the number of helminth eggs at each WWTP. The total number of viable helminth eggs from the Águas Lindas de Goiás (AGL) WTTP was compared to the other WWTPs in the Federal District (BSN—Brasília Norte; BSS—Brasília Sul; GAM—Gama; MLC—Melchior; RCE—Recanto das Emas; RF1—Riacho Fundo; SB1—Sobradinho). Statistical significance was assessed by one-way ANOVA to compare median values among the different groups, followed by Tukey’s multiple comparisons test. *P = 0.0195; **P = 0.007; ***P = 0.0003; ****P = 1.318 × 10⁻⁷;* P* = 2.13 × 10⁻⁸, and *P* = 8.8 × 10⁻⁹, respectively

Parasitological analysis of the sewage sludge samples revealed significant variations in helminth egg concentration among the three socioeconomic groups (low-, medium-, and high-income regions). The highest average number of viable helminth eggs was observed in the low-income category, specifically at AGL, where the mean count reached 16.61 ± 3.02 eggs/g DM (Fig. 5). This value is approximately five times higher than that observed at the Brasília Norte WWTP (BSN; 3.559 ± 0.5464 eggs/g DM; *P* = 8.8 × 10^–9^), which serves a high-income population. Notably, AGL, a city with over 225,000 inhabitants [24], has an average monthly income about six times lower than that of residents in areas served by BSN (Table 1).

The highest helminth egg concentrations in the Federal District were observed in the west and southwestern regions, specifically at the Gama (10.73 ± 1.54 eggs/g DM), Recanto das Emas (10.07 ± 1.08 eggs/g DM), Melchior (9.64 ± 1.23 eggs/g DM), and Riacho Fundo (7.25 ± 1.23 eggs/g DM) WWTPs. These facilities primarily serve medium-income regions. There were no significant differences in helminth egg counts among these WWTPs (*P* = 0.3875). However, compared to the WWTP in Goiás, which serves low-income areas, GAM was the only one that did not show a statistically significant difference (*P* = 0.06; Fig. 5). Interestingly, although the minimum wage in Sobradinho WWTP (SB1) is comparable to other medium-income regions, the number of viable helminth eggs detected in SB1 (4.61 ± 0.61 eggs/g DM) was significantly lower (*P* = 0.0015). This discrepancy may be attributed to differences in population density, local environmental conditions, or variations in sanitation infrastructure effectiveness.

**Fig. 6:**
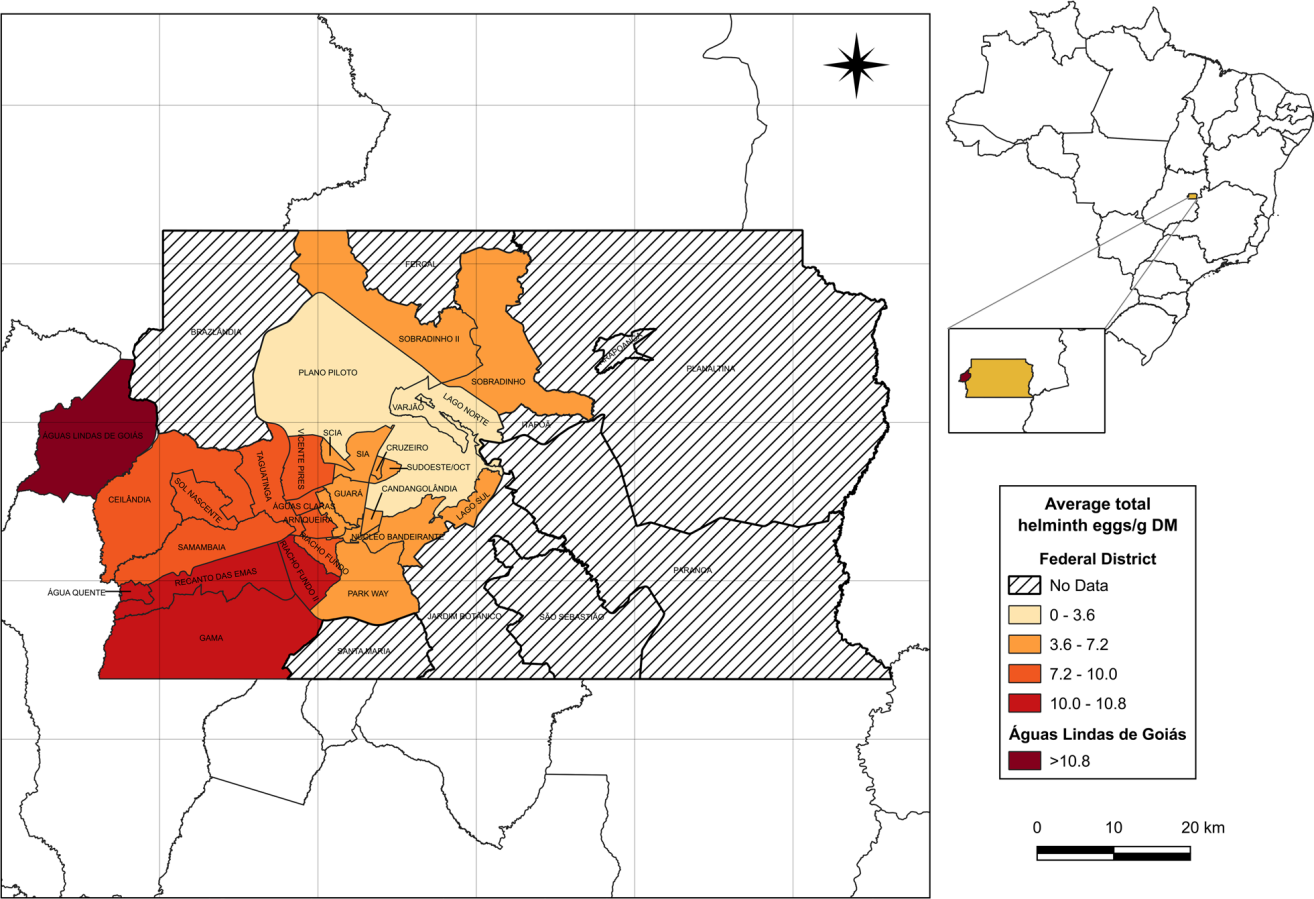
Map of the total viable helminth eggs distribution in the Federal District and Águas Lindas de Goiás. Locations classified as "no data" are not covered by the analyzed WWTPs

In contrast, high-income regions (BSN and BSS) had the lowest helminth egg loads, with mean values of 3.56 ± 0.55 eggs/g DM and 3.90 ± 0.67 eggs/g DM, respectively. These WWTPs serve areas with the highest average monthly incomes, including the central region of Brasília (Fig. 6), where sanitation infrastructure is more developed and access to clean water is nearly universal. This region, known as the Pilot Plan of Brasília, houses the country's main political-administrative buildings. The differences between high- and low-income regions were statistically significant (*P* = 2.13 × 10⁻⁸ and *P* = 8.8 × 10⁻⁹ for BSS and BSN, respectively), highlighting the impact of socioeconomic factors on helminth prevalence.

## Discussion

Viable helminth eggs were identified and quantified in sewage sludge samples from eight WWTPs in the Federal District and Goiás. The results revealed a predominance of *Hymenolepis* spp. eggs, a cestode that infects at least 50–75 million people worldwide [33]. *H. nana*, the most prevalent cestode in humans, presents a direct infection cycle, whereas *H. diminuta* requires ingestion of infected insects and typically involves rodents as definitive hosts [34]. Brazil is among the three countries with the highest number of *H. diminuta* cases [35]. In previous analyses of sewage sludge from São Paulo [26] and Paraná [25], *Hymenolepis* spp. eggs were the third most frequent (7.68% and 4.67%, respectively), while *Ascaris* sp*.* and *Toxocara* sp*.* eggs ranked first and second, respectively. The findings of this study suggest a high occurrence of hymenolepiasis throughout the Brazilian capital, predominantly in medium- and high-income regions. This divergence in the predominance of *Hymenolepis* spp*.* may be associated with underdiagnosed zoonotic cycles or foodborne transmission, particularly in urban and peri-urban areas. Meanwhile, in low-income regions, *Ascaris* spp. and *Trichuris* spp. were more prevalent, both of which are highly resistant to environmental stressors and frequently associated with open defecation and inadequate sanitation [4, 11].

Our findings align with broader sewage sludge analyses conducted in Brazil [36, 37] and abroad [38–42], where *Ascaris* sp., *Trichuris* sp., and *Toxocara* sp. eggs dominate. *Ascaris* sp. eggs, viable or not, are commonly reported in sewage sludge analysis [25, 26, 38]. However, this study focused exclusively on viable eggs. Due to their pathogenicity, specific gravity, prevalence, and high resistance to environmental factors, *Ascaris* spp., *Trichuris* spp., and *Toxocara* spp. eggs are commonly used as key indicators of sewage treatment efficiency [43–45].

Infected individuals can excrete 10^2^–10^4^ eggs/g daily, contributing to high concentrations of helminth eggs in sewage sludge [46]. The frequent occurrence of helminthic polyparasitism in humans was previously reported in various endemic regions [47–51]. Intestinal pathogen associations can manifest in both antagonistic and synergistic ways. Previous studies have shown that the simultaneous presence of *A. lumbricoides* and hookworms can increase the number of eggs produced by both species [52]. Individuals infected with more than one helminth commonly present more severe infections, with intensified symptoms such as anemia [51, 53, 54]. Moreover, helminths often co-occur with viruses and protozoa, influencing susceptibility to infection and impacting disease progression in malaria, leishmaniasis, and HIV infection [1, 55–59]. Notably, *Ascaris* spp., *Trichuris* spp., and *Schistosoma* spp., commonly related to *Leishmania* spp. and HIV coinfection [55, 56, 60], were detected in this study at high incidence rates (19.27%, 7.87%, and 5.25%, respectively).

Our study provides direct epidemiological evidence supporting the well-established association between socioeconomic inequality and helminth infections. A clear gradient was observed, with significantly higher egg concentrations in sewage sludge from low-income regions. Similar patterns have been reported in Brazil and globally, where poverty, limited education, and poor sanitation consistently elevate infection risks [61–64]. Countries with a per capita GDP exceeding USD 20,000 exhibit minimal or no infection [61]. In addition to socioeconomic influences, our findings also highlight regional disparities in helminth species composition, suggesting variations in environmental exposure and transmission dynamics. The predominance of nematodes in low-income areas suggests inadequate sanitation, whereas the higher prevalence of *Hymenolepis* spp. in medium- and high-income regions indicates a greater influence of zoonotic or foodborne transmission pathways. Therefore, while socioeconomic inequality is a major determinant of STH prevalence, local environmental and behavioral factors also play a crucial role in shaping infection patterns.

WBE is a non-invasive epidemiological tool that enables the simultaneous monitoring of large populations, including asymptomatic individuals, thereby indicating potential transmission hotspots and facilitating early detection of outbreaks [65]. However, this approach has limitations, primarily due to the absence of standardized methods for detecting helminth eggs in sewage sludge [66]. Flotation techniques, such as the one used in this study, are the most employed, with the use of solutions ranging in density from 1.20 to 1.30 g/ml recommended to recover the majority of the eggs [43], considering that most eggs have specific gravities between 1.05 and 1.23 [67]. Nevertheless, the relative density of many helminth eggs is still unknown, and eggs with specific gravities very close to the solution density may not be fully recovered during the process. *Taenia* sp. eggs are an example, as they have a high specific gravity (SG = 1.2251) [67]. Moreover, the 21–28 day incubation period may not be suitable for all parasites, as some larvae can hatch early and die before detection. Chemicals additives used in sewage sludge dewatering process, such as polyelectrolytes, can hinder detection by increasing egg adhesion to solids. Furthermore, because each WWTP serves a broad area, the spatial resolution of results is limited. Despite these limitations, WBE remains valuable for environmental and epidemiological surveillance of underreported and neglected infections.

## Conclusions

This study identified and quantified helminth eggs found in sewage sludge from WWTPs in socioeconomically diverse regions of the Federal District and Goiás, located in Brazil’s central-west region. Eggs from 12 distinct genera were identified, including five nematodes and seven platyhelminths. The parasitological profile of the sewage sludge samples indicated a high incidence of cestode flatworms, with a predominance of *Hymenolepis* spp., which constitute nearly half of the detected eggs. Our findings demonstrate that regions with lower average incomes had more significant quantities of helminth eggs, emphasizing the impact of socioeconomic factors on the prevalence of helminthiases. Through the parasitological description of the sewage sludge from each WWTP, potential hotspots for certain helminthiases can be suggested, contributing to the epidemiological surveillance of these parasitic diseases. Continuous surveillance would facilitate the systematic tracking of infection trends over time, assisting in the identification of fluctuations in the prevalence of helminthiases in the different regions analyzed.

## Data Availability

The datasets used and/or analysed during the current study are available from the corresponding author on reasonable request.

## Abbreviations

BOD: Biochemical oxygen demand
BSN: Brasília Norte
BSS: Brasília Sul
CAESB: Environmental Sanitation Company of the Federal District
CMAL: Complete-Mix Aerated Lagoon
DM: Dry Mass
GAM: Gama
MLC: Melchior
MW: Minimum wage
RCE: Recanto das Emas
RF1: Riacho Fundo
SB1: Sobradinho
SG: Specific gravity
STH: Soil-transmitted helminths
UASB: Upflow Anaerobic Sludge Blanket
WBE: Wastewater-based epidemiology
WWTP: Wastewater treatment plant
